# Association between capillary congestion and macular edema recurrence in chronic branch retinal vein occlusion through quantitative analysis of OCT angiography

**DOI:** 10.1038/s41598-021-99429-z

**Published:** 2021-10-06

**Authors:** Min Seung Kang, Sang Yoon Kim, Sung Who Park, Ik Soo Byon, Han Jo Kwon

**Affiliations:** 1 Department of Ophthalmology, Pusan National University Yangsan Hospital, Geumo-ro 20, Mulgeum-eup, Yangsan-si, Gyeongsangnam-do 50612 South Korea; 2 Research Institute for Convergence of Biomedical Science and Technology, Pusan National University Yangsan Hospital, Geumo-ro 20, Mulgeum-eup, Yangsan-si, Gyeongsangnam-do 50612 South Korea; 3 Pusan National University School of Medicine, Geumo-ro 20, Mulgeum-eup, Yangsan-si, Gyeongsangnam-do 50612 South Korea; 4 Department of Ophthalmology, Biomedical Research Institute, Pusan National University Hospital, Gudeok-ro 179, Seo-gu, Busan, 49241 South Korea

**Keywords:** Retinal diseases, Risk factors, Prognostic markers

## Abstract

This study aims to quantitatively investigate the optical coherence tomographic angiography (OCTA) findings of capillary congestion and its association with macular edema (ME) recurrence in chronic branch retinal vein occlusion (BRVO). We retrospectively reviewed the medical records of 115 consecutive patients with major ischemic BRVO who reached stable macula (without ME for two consecutive visits) at baseline (the first visit within the stable period). All patients were classified into a recurrence or non-recurrence groups depending on ME recurrence. Capillary congestion of deep capillary plexuses (DCP-C) and other abnormal capillary lesions were segmented, and their areas, vascular densities, and mean retinal thicknesses (MRT) were calculated. The main outcomes were differences between the two groups and risk factors for recurrence among baseline and OCTA parameters. A total of 76 eyes were included, of which 22 (28.9%) recurred. DCP-C existed in all eyes at baseline. MRT of DCP-C (p = 0.006) was greater in the recurrence group. Greater MRT of DCP-C (OR: 1.044; p = 0.002) and more frequent intravitreal injections (OR: 1.803; p < 0.001) were associated with a higher risk of relapsing ME. DCP-C may contribute to the anatomical stability of chronic BRVO and simultaneously be the source of ME.

## Introduction

Retinal vein occlusion (RVO) is the second most common retinal vascular disease, and macular edema (ME) is the leading cause of visual loss in patients with RVO^1^. RVO can be subdivided into central, hemispheric, and branch retinal vein occlusion (BRVO)^2^. Acute BRVO presents with retinal hemorrhages, dilated tortuous veins, cotton wool spots, and ME through increased venous pressure from the obstructed arteriovenous junction to its distal compartment^1,3^. Chronic ME, macular ischemia, and complications related to retinal neovascularization lead to irreversible visual impairment after acute BRVO^3–6^. Nevertheless, without any treatment, visual acuity improves by two or more lines in up to three-quarters of patients with acute BRVO, and ME in acute BRVO resolves in up to 41% of cases after 7.5 months^7^. Few studies have been conducted to clarify how chronic BRVO sustains a stable macula and which clinical parameters increase the risk of ME recurrence in chronic BRVO.

Although arteriovenous occlusion is not restored and venous return is continuously impeded in chronic BRVO, the macula often maintains anatomical stability without edema and simply presents with sclerotic veins and venous collaterals^1,8^.

Optical coherence tomographic angiography (OCTA) can better characterize the abnormal vascular lesion by splitting it into superficial and deep capillary plexuses (SCP and DCP) without contrast agents. Disruption of the perifoveal capillary network, capillary nonperfusion area (NPA), microaneurysms, retinal neovascularization, and capillary congestion reflected by OCTA have been reported as representative abnormal vascular changes in BRVO^9^. Among them, capillary congestion on OCTA has been observed as dilation of the capillary network with hyper-signal, capillary telangiectasia, and vascular dilation^10–13^. In addition, capillary congestion has been studied in terms of collateral vessels and venous collaterals with fluorescein angiography (FA)^14–16^. Freund and colleagues found that these dilated and tortuous collateral vessels detected in BRVO were mainly distributed in the intermediate capillary plexus and DCP on OCTA^17^. Capillary congestion was well depicted at the boundaries of normal and abnormal capillary plexuses in chronic BRVO rather than acute BRVO and on OCTA rather than FA^10–13,16,18^. Interestingly, capillary congestion appeared primarily in DCP as collateral vessels shown in FA in all patients with BRVO^11,13^.

Capillary loss only in DCP was associated with persistent ME in BRVO^19^, but little is known about the anatomic features and clinical significance of capillary congestion or abnormal vascular lesions in chronic BRVO before ME recurrence. Considering the coexisting fluids on OCTA images resulting in inaccuracies and segmentation errors and also the efforts to correct them, most research on the association between OCTA parameters and BRVO has focused on qualitative features of capillary congestion^9^.

Here, we performed a clinical study based on quantitative analysis of OCTA to investigate the spatial distribution of capillary congestion with other abnormal vascular lesions and its association with recurrence of ME in chronic BRVO.

## Methods

### Patient selection

**Figure 1:**
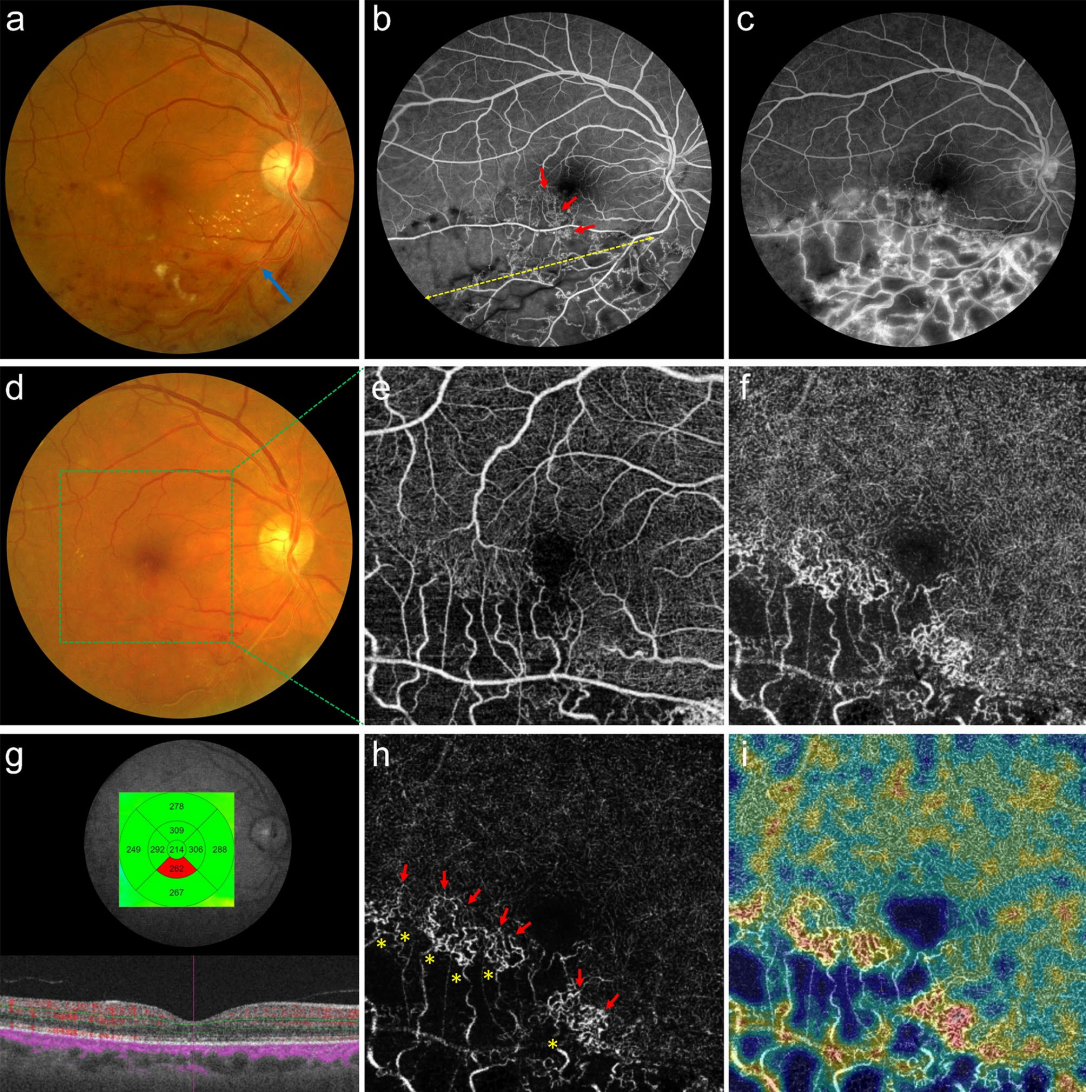
Patient selection and capillary congestion at baseline. (**a**) A patient presented with acute major branch retinal vein occlusion (BRVO) at the right inferotemporal arteriovenous crossing (blue arrow). (**b**, **c**) Three months after the initial visit, a wide capillary nonperfusion area (yellow dotted double arrow) is shown on fluorescein angiography (FA), confirming ischemic BRVO. FA presents collateral vessels (red arrows) with fluorescein leakage at the late phase. (**d**) He reached stable macula at baseline 20 months after the initial visit with seven anti-vascular endothelial growth factor intravitreal injections and sectorial photocoagulation. (**e**, **f**) At baseline, en-face optical coherence tomographic angiography corresponding to the green box area reveals the nonperfusion areas on superficial and deep capillary plexuses (DCP). (**g**) The central subfield macular thickness was 214 µm without any fluids at baseline. (**h**) The gamma was adjusted to clarify the capillary congestion of DCP (DCP-C) from the (**f**) image. The retinal arterioles (yellow asterisks) from the nonperfusion area supply flow to DCP-C, and the flow drains to the central vortex venules on the opposite side (red arrows). (**i**) Abundant flows of DCP-C were noted on the composite image of en-face DCP and the color-coded vessel density map.

We reviewed the medical records of 115 consecutive patients who met the inclusion criteria and visited the outpatient clinic of the ophthalmology department between January 2017 and December 2019. The major inclusion criterion was only major BRVO, except for macular BRVO according to Hayreh's classification^20^. The other inclusion criteria were unilateral, acute, and ischemic BRVO with maintained chronic stable macula 6 months after from the initial visit. All patients had undergone swept-source optical coherence tomography (SS-OCT, DRI OCT-1 Atlantis; Topcon Corp., Tokyo, Japan) and OCTA with the same OCT device at each visit. At the initial visit, all patients received a diagnosis based on the clinical findings of acute BRVO detected on fundus photography. Three months after the first visit, ischemic BRVO was evaluated as the existence of NPA more than five times larger than the optic disc diameter on FA (Fig. 1)^21^.

All cases of ME secondary to BRVO were treated with the pro re nata regimen without an initial anti-vascular endothelial growth factor (VEGF) loading dose. Initial anti-VEGF treatment or retreatment was performed if at least one of the following two criteria was met: CSMT greater than 300 μm accompanied by intraretinal or subretinal fluid, or decline in BCVA of two or more lines due to intraretinal or subretinal fluid compared with the previous visit. An intravitreal steroid injection was allowed if the retreatment criteria were satisfied even after two consecutive anti-VEGF injections.

Stable macula was defined as the anatomical conditions of the macula without retinal hemorrhages, cotton wool spots, and ME for two successive visits with a 3-month interval. The baseline was defined as the first visit during the period while the chronic stable macula was maintained (see Supplementary Fig. S1). Thirty-nine patients who had met the inclusion criteria were excluded from the study based on the exclusion criteria summarized in Supplementary Table S1.

### Data collection

The following factors were investigated as baseline characteristics: age, sex, laterality, occlusion site, best-corrected visual acuity (BCVA) and central subfield macular thickness (CSMT) at baseline, medical history (hypertension, diabetes mellitus, or dyslipidemia) diagnosed by internal medicine specialists, treatment history (intravitreal injections and sectorial scatter photocoagulation) up to baseline, and the period from the initial visit to baseline. At each visit, two ophthalmologists (M.S.K. and S.Y.K.) assessed ME using fovea-centered 6 × 6 mm^2^ macular volume scans acquired with SS-OCT. The absence of ME during the period from the initial visit to baseline was determined if CSMT did not exceed 300 μm without intraretinal or subretinal fluids^22^. From baseline to 12 months, recurrence was assessed using the same modalities of the OCT device. Eyes with CSMT that increased by 10% or more from baseline due to any fluids from the baseline were classified as the recurrence group, and the remaining eyes were classified as the non-recurrence group. In the recurrence group, CSMT at recurrence and the period from baseline was investigated.

### Acquisition of En-face optical coherence tomographic angiography at baseline

For acquisition of en-face OCTA images, a fovea-centered 6 × 6 mm^2^ macula region was scanned. With a 1050 nm swept-source laser and a 100 k A-scans/s speed, the device detected moving flows based on the OCTA Ratio Analysis algorithm. SCP images were derived from two en-face slabs, placed between 2.6 μm posterior to the internal limiting membrane and 15.6 μm posterior to the junction of the inner plexiform and inner nuclear layers (IPL and INL). DCP images were obtained by applying slabs from 15.6 to 70.2 μm posterior to the intersection of the IPL and INL. Each OCTA image was stored as a 16-bit gray-scale image with a resolution of 600 × 600 pixels. The color-coded retinal thickness and vascular density (VD) maps on the same scan area were simultaneously provided by the built-in software (IMAGEnet 6 ver. 1.24; Topcon Corp., Tokyo, Japan) and saved as 24-bit color images with the same pixel resolution. The self-made conversion software using the Python programming language (Python ver. 3.7.7; Python Software Foundation, Wilmington, DE, USA) converted the color maps into 16-bit gray-scale images based on the color conversion code provided by the manufacturer (see Supplementary Fig. S2).

### Manual segmentation and quantitative analysis of abnormal vascular areas

With the use of a commercial image processing program (Photoshop CS3 ver. 10.0; Adobe Systems, Mountain View, CA, USA), capillary NPA, aneurysmal dilatation, and capillary congestion except for the foveal avascular zone (FAZ) of each layer were selected as abnormal regions. By designating the pixel values of an abnormal region as TRUE and the normal capillaries as FALSE, we separately generated binary images of an abnormal region of SCP (SCP-A) and DCP (DCP-A). The inverses of SCP-A and DCP-A except for the FAZ were labeled as the normal region of SCP (SCP-N) and DCP (DCP-N), respectively. In addition, segmentation images for FAZ were produced by selecting the FAZ of SCP and DCP (SCP-FAZ and DCP-FAZ). The transitional zone (TZ) was defined as the region of abnormal DCP under normal SCP and was automatically calculated by the Hadamard product of two binary matrices, SCP-N and DCP-A.

**Figure 2:**
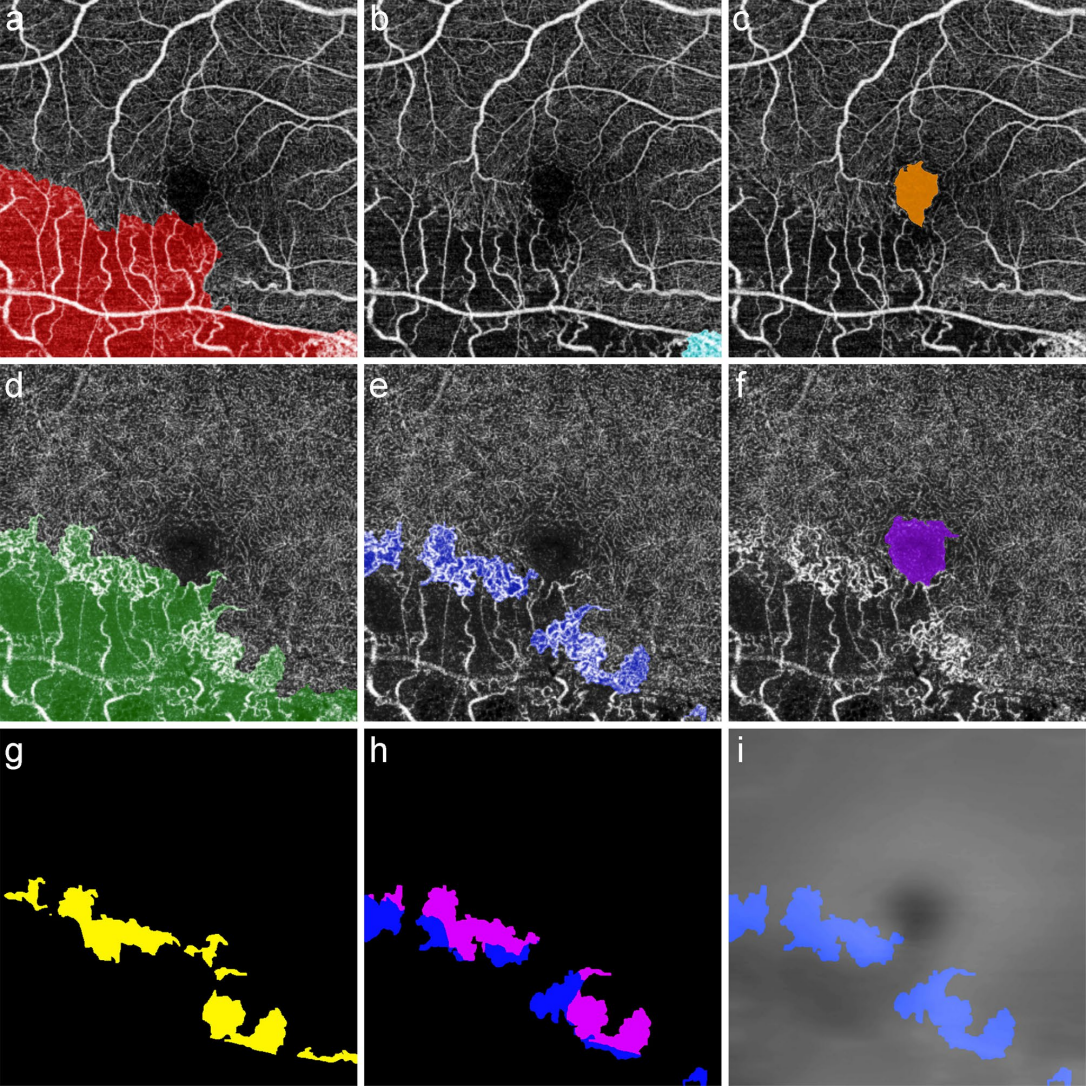
Segmentation of capillary congestion, abnormal vascular lesion, and foveal avascular zone in each capillary plexus. Segmentation process for the same eye shown in Fig. 1. (**a**) Red indicates an abnormal vascular lesion of the superficial capillary plexus (SCP-A). (**b**) Cyan indicates capillary congestion of the SCP. (**c**) Orange indicates the foveal avascular zone (FAZ) of the SCP. (**d**) Green indicates an abnormal vascular lesion of the deep capillary plexus (DCP-A). (**e**) Blue indicates capillary congestion of the DCP (DCP-C). (**f**) The purple region is the FAZ of the DCP. Ophthalmologists saved these six sets of manual segmentation data as 600 × 600 size of two-dimensional binary matrices composed of TRUE or FALSE. Colored pixels were assigned to TRUE and colorless pixels to FALSE. (**g**) DCP-A was larger than SCP-A, and the transitional zone (TZ, yellow) was formed in the region of abnormal DCP under normal SCP. (**h**) The DCP-C image was superimposed on the TZ. The pink-colored region indicates DCP-C comprised in the TZ, and 45.0% of DCP-C was spread in the TZ. (**i**) The gray-scaled retinal thickness map was superimposed on the segmented DCP-C image. The mean retinal thickness of the DCP-C (blue region) was calculated to be 276.1 µm at baseline.

We defined the region of the capillary congestion of SCP (SCP-C) and DCP (DCP-C) as expanded, bright, and coarse capillary networks between the affected and normal vasculatures. From quantitative analysis of capillary diameter using confocal microscopy, normal capillary diameter ranged from 5 to 13 μm, and mean capillary diameter did not differ between layers^23^. Considering that one pixel of the acquired en-face OCTA image has a resolution of 10 μm (600 pixels per 6.0 mm), en-face OCTA can determine the normal retinal capillary diameter within two pixels. On the basis of this evidence, an expanded capillary was defined as a capillary with a diameter exceeding two pixels in each en-face OCTA image; a bright capillary was defined as a capillary with a gray level greater than 50% (e.g., pixel values ≥ 32,768 in a 16-bit gray-scale image); and a coarse capillary was defined as a capillary with a diameter that fluctuated by more than two pixels until it was connected to adjacent capillaries. These networks receive arterial flows from the precapillary arterioles of the occlusive side and drain into the postcapillary venules of the unaffected side. To identify the boundary of capillary congestion, gamma correction (γ = 3.0) was applied to en-face OCTA images (Fig. 1h). Only the capillary congestion was assigned to TRUE, so binary images of SCP-C and DCP-C could be extracted (Fig. 2). M.S.K. and S.Y.K. performed all six binarization processes (SCP-A, DCP-A, SCP-C, DCP-C, SCP-FAZ, and DCP-FAZ), and inconsistent boundaries were finally resolved by H.J.K. Segmented area, mean VD, and mean retinal thickness (MRT) corresponding to the segmented regions were automatically calculated using a self-made software coded on Python.

### Spatial distribution of deep capillary congestion and macular edema in the recurrence group

**Figure 3:**
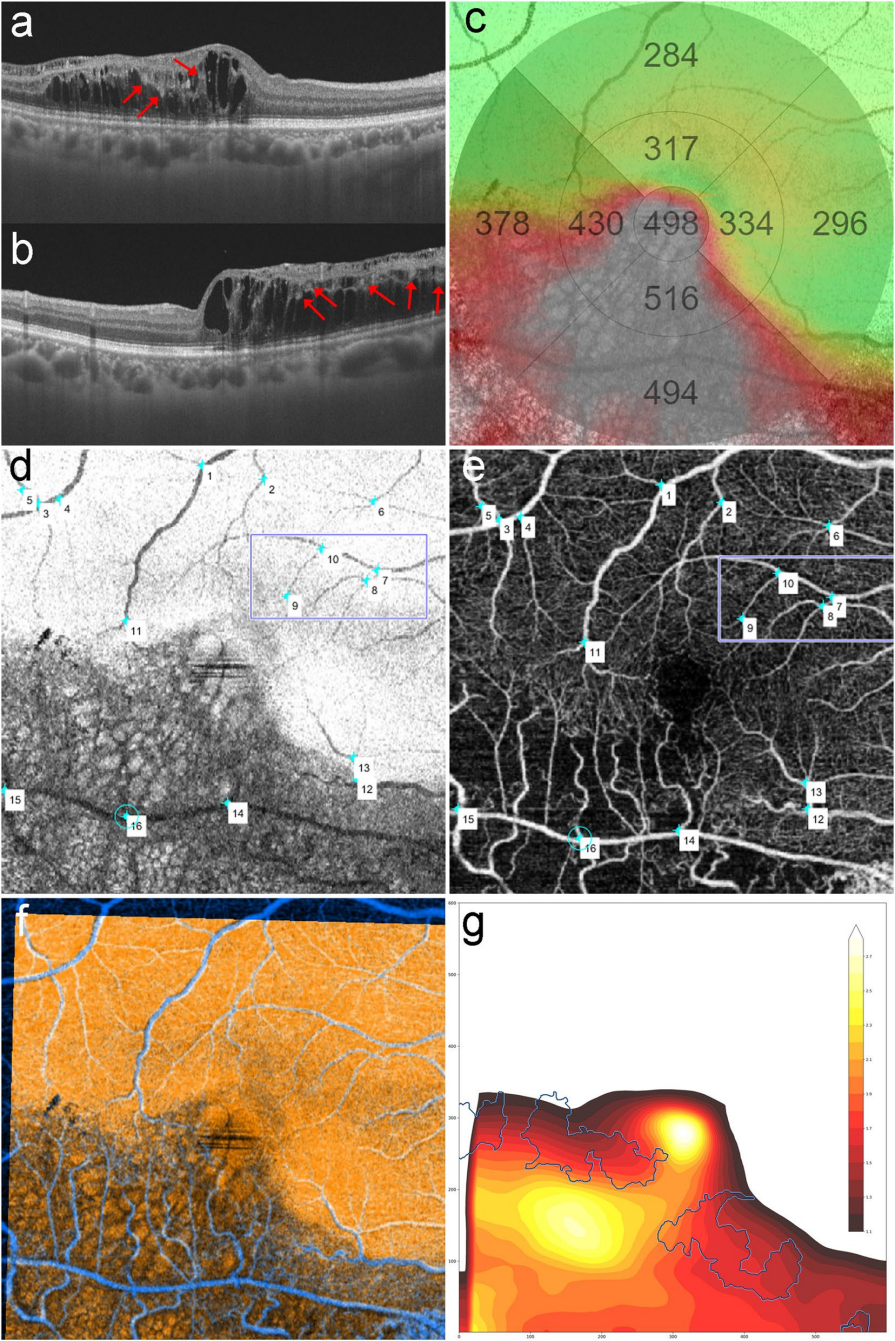
Alignment of en-face optical coherence tomography at recurrence to baseline en-face optical coherence tomographic angiography to visualize the territory of macular edema. (**a**–**c**) The patient noted in Fig. 2 showed macular edema (ME) recurrence at 6 months after baseline. Red arrows indicate capillary congestion of the deep capillary plexus (DCP-C) surrounded by ME in horizontal (**a**) and vertical (**b**) B-scan optical coherence tomography (OCT). In quantitatively analyzing the ratio of increased retinal thickness (RIRT) on a pixel-by-pixel basis, an alignment is required to correct for relative eye movement between two visits. (**d**) Sixteen control points were selected at the apparent bifurcation of the en-face OCT image at the recurrence. (**e**) Superficial capillary plexus OCT angiography (OCTA) at baseline presents selected control points at the same bifurcation. Matlab software calculated the geometric relationship between two images in a 3 × 3 affine transformation matrix through the coordinates of two sets of control points. (**f**) The color of the en-face OCT was designated as orange after the transformation. The OCTA at baseline was designated as blue and merged with the transformed en-face OCT image to confirm the alignment. By applying the same matrix to the macular thickness color map at recurrence, it is possible to analyze the RIRT in pixel units. (**g**) The RIRT of each pixel was calculated, and the territory of ME was plotted with a contour plot. Python software assigned different colors to pixels whose ratio increased by 1.1 or more (assigned colors are indicated in a color bar at the upper right corner). Then, the boundary of DCP-C was covered with blue lines. The maximum RIRT was 2.833 at the fovea; DCP-C was juxtaposed with the ME territory and two local maxima.

To depict the ratio of increased retinal thickness (RIRT) at a pixel level between baseline and recurrence, an en-face OCT image from macular volume scan at recurrence and an en-face SCP image at baseline were aligned. Using the Control Point Selection Tool of Matlab (Matlab R2018a; The Mathworks Inc, Natick, MA, USA), bifurcations in each image were manually selected (by H.J.K.), and the geometric transformation was solved (Fig. 3). After aligning two provided retinal thickness maps from macular volume scan and OCTA with affine transformation, the RIRT on the overlapping area was obtained. The ME territory was defined as the boundary of the RIRT with more than a 10% increase from baseline and was presented with a contour plot using Python software. Finally, the DCP-C boundary was superimposed on the contour plot to visualize the distribution of DCP-C and the ME territory. The average and maximum RIRT of DCP-C were compared with those of the ME territory. In addition, we investigated the location of the apex of RIRT in the ME territory.

### Main outcome measures and statistical analyses

The main outcome measures were the differences between the two groups and risk factors for the recurrence of ME among baseline characteristics and OCTA parameters. BCVA was measured with a Snellen chart and converted to the logarithm of the minimum angle of resolution (logMAR) for statistical analysis. Statistical analysis software (SPSS ver. 21.0; SPSS Inc., Chicago, IL, USA) was used for comparison of the two groups; specifically, the Mann–Whitney *U* test, the Wilcoxon signed-rank test, and the Pearson chi-squared test with Fisher's exact probability test were applied for evaluation of independent numerical data, dependent numerical data, and categorical data, respectively. Univariate and multivariate binary logistic regression analysis was employed to identify the factors affecting the recurrence of ME. p values < 0.05 were considered to indicate statistical significance.

### Ethics approval

 The authors conducted this retrospective, comparative, and observational study under the ethical standards set out in the Declaration of Helsinki. Informed consent was obtained from all participants. The institutional review board of Pusan National University Yangsan Hospital approved the protocol of the study (approval no. 05-2020-262).

## Results

This study included 76 eyes with chronic stable BRVO, of which 22 (28.9%) showed recurrence of ME. Table 1 summarizes the baseline characteristics of the two groups and their comparisons. The mean age of the recurrence group was greater than that of the non-recurrence group at baseline (67.8 vs. 61.8 years, p = 0.028). At baseline, mean BCVA was 0.218 logMAR and CSMT was 226.4 μm; no differences were observed between the two groups. The mean period from the initial visit to baseline was 16.1 ± 7.0 months and was significantly longer in the recurrence group (20.9 vs. 14.1 months, p < 0.001). The recurrence group required more intravitreal injections (5.8 vs. 2.4, p < 0.001), more intravitreal anti-VEGF injections (3.6 vs. 2.0, p = 0.002) and steroid (2.2 vs. 0.4, p = 0.005) injections. Representative cases of the non-recurrence and recurrence group are shown in Supplementary Fig. S3.

**Table 1:** Baseline characteristics of patients with chronic branch retinal vein occlusion.

	Total group N = 76	Non-recurrence group N = 54	Recurrence group N = 22	p value
Age (years)	63.5 ± 10.9	**61.8 ± 9.8**	**67.8 ± 12.3**	**0.028**^**a**^
Male/female (N)	38/38	26/28	12/10	0.610^b^
Right/left (N)	39/37	24/30	15/7	0.060^b^
Hypertension (N)	48	34	14	1.000^b^
Diabetes mellitus (N)	10	5	5	0.142^c^
Dyslipidemia (N)	5	5	0	0.313^c^
Period from initial visit to the baseline (months)	16.1 ± 7.0	**14.1 ± 5.3**	**20.9 ± 8.3**	**< 0.001**^**a**^
Mean baseline BCVA (logMAR)	0.218 ± 0.241	0.187 ± 0.216	0.295 ± 0.284	0.138^**a**^
Mean baseline CSMT (μm)	226.4 ± 33.3	224.1 ± 32.8	232.2 ± 34.7	0.379^**a**^
Period from initial visit to first anti-VEGF injection (days)	9.5 ± 5.8	9.4 ± 6.0	9.6 ± 5.5	0.706^**a**^
**Total number of intravitreal injections (N)**	3.4 ± 3.2	**2.4 ± 2.4**	**5.8 ± 3.7**	**< 0.001**^**a**^
Anti-VEGF (N)	2.5 ± 2.1	**2.0 ± 1.9**	**3.6 ± 2.2**	**0.002**^**a**^
Steroid (N)	0.9 ± 2.3	**0.4 ± 0.9**	**2.2 ± 3.8**	**0.005**^**a**^
Sectorial scatter photocoagulation (N)	56	40	16	0.920^b^
**Occlusion site**				
Superotemporal branch (N)	54	41	13	0.143^b^
Inferotemporal branch (N)	22	13	9	

*BCVA* best-corrected visual acuity, *CSMT* central subfield macular thickness, *VEGF* vascular endothelial growth factor.

Bold font indicates a statistically significant difference (a p value < 0.05) between the non-recurrence and recurrence groups.

^a^The results of the Mann–Whitney *U* test.

^b^The results of Pearson chi-squared test.

^c^The results of the Fisher's exact probability test.

Laser photocoagulation did not affect baseline BCVA, CSMT, or ME recurrence. In patients treated with sectorial photocoagulation, the areas of the SCP-A (9.935 vs. 7.653 mm^2^, p = 0.044) and the DCP-A (14.039 vs. 11.501 mm^2^, p = 0.017) were greater, and total SCP VD (41.3 vs. 43.1%, p = 0.004), SCP-A VD (35.7 vs. 39.1%, p = 0.003), and total DCP VD (42.5 vs. 43.5%, p = 0.031) were lower, but other OCTA parameters did not differ between the groups.

### Area of capillary congestion with other abnormal vascular lesions

Table 2 shows the mean areas of the segmented regions at baseline. DCP-A was greater than SCP-A in all of the investigated eyes (p < 0.001). SCP-C was shown in 67 eyes (88.2%), and the frequency did not differ between the two groups. However, DCP-C existed in all of the eyes. Fifty-seven percent of DCP-C area was in the TZ, and the rest was in the DCP-A under SCP-A (1.455 vs. 1.077 mm^2^, p < 0.001). The mean area of the SCP-FAZ was smaller than that of the DCP-FAZ (0.431 vs. 0.641 mm^2^, p < 0.001). None of the investigated areas differed between the two groups.

**Table 2:** Mean area corresponding to the segmented regions at baseline.

	Total group N = 76	Non-recurrence group N = 54	Recurrence group N = 22	p value^a^
**Mean area**				
SCP-A area (mm^2^, %)	9.334 ± 4.514 (25.9 ± 12.5%)	8.957 ± 4.677 (24.9 ± 13.0%)	10.261 ± 4.039 (28.5 ± 11.2%)	0.238
DCP-A area (mm^2^, %)	13.371 ± 4.357 (37.1 ± 12.1%)	12.784 ± 4.454 (35.5 ± 12.4%)	14.810 ± 3.830 (41.1 ± 10.6%)	0.083
TZ area (mm^2^, %)	4.279 ± 3.199 (11.9 ± 8.9%)	4.060 ± 2.897 (11.3 ± 8.0%)	4.814 ± 3.867 (13.3 ± 10.7%)	0.498
SCP-C area^b^ (mm^2^, %)	0.716 ± 0.849 (2.0 ± 2.4%)	0.648 ± 0.906 (1.8 ± 2.5%)	0.882 ± 0.680 (2.4 ± 1.9%)	0.056
DCP-C area (mm^2^, %)	2.532 ± 0.952 (7.0 ± 2.6%)	2.475 ± 0.931 (6.9 ± 2.6%)	2.672 ± 1.009 (7.4 ± 2.8%)	0.456
DCP-C area in TZ (mm^2^, %)	1.455 ± 0.775 (57.5 ± 30.6%)	1.417 ± 0.714 (57.3 ± 26.7%)	1.549 ± 0.918 (58.0 ± 34.4%)	0.923
DCP-C area under SCP-A (mm^2^, %)	1.077 ± 0.718 (42.5 ± 28.4%)	1.058 ± 0.712 (42.7 ± 26.6%)	1.123 ± 0.749 (42.0 ± 28.0%)	0.662
SCP-FAZ area (mm^2^, %)	0.431 ± 0.173 (1.2 ± 0.5%)	0.422 ± 0.181 (1.2 ± 0.5%)	0.453 ± 0.154 (1.3 ± 0.4%)	0.307
DCP-FAZ area (mm^2^, %)	0.641 ± 0.358 (1.8 ± 1.0%)	0.622 ± 0.354 (1.7 ± 1.0%)	0.690 ± 0.373 (1.9 ± 1.0%)	0.422

*SCP-A* abnormal region of superficial capillary plexus, *DCP-A* abnormal region of deep capillary plexus, *TZ* transitional zone, *SCP-C* capillary congestion of superficial capillary plexus, *DCP-C* capillary congestion of deep capillary plexus, *FAZ* fovea avascular zone, *SCP-FAZ* foveal avascular zone of superficial capillary plexus, *DCP-FAZ* foveal avascular zone of deep capillary plexus.

Bold font indicates a statistically significant difference (a p value < 0.05) between the non-recurrence and recurrence groups.

^a^All values are the results of the Mann–Whitney *U* test.

^b^Statistical values for 67 eyes (47 eyes in the recurrence group and 20 eyes in the non-recurrence group) in which SCP-C was observed.

### Mean vascular density and mean retinal thickness of the segmented regions

Table 3 shows the mean VD, and MRT of the segmented regions at baseline. The mean VD of SCP-A was lower than that of SCP-N (36.6 vs. 44.3%, p < 0.001) in all of the eyes; the mean VD of SCP-C was higher than those of SCP-A and SCP-N (all, p < 0.001). Similarly, the mean VD of DCP-A was lower than that of DCP-N (40.3 vs. 44.9%, p < 0.001), and the mean VD of DCP-C was higher than those of DCP-A and DCP-N in all eyes (all, p < 0.001). However, there were no differences between the two groups in the mean VD in any of the segmented regions.

**Table 3:** Mean vascular density and retinal thickness of the segmented regions.

	Total group N = 76	Non-recurrence group N = 54	Recurrence group N = 22	p value^a^
**Mean vascular density of SCP**				
Total SCP VD (%)	41.8 ± 2.5	41.8 ± 2.6	41.6 ± 2.3	0.556
SCP-A VD (%)	36.6 ± 4.9	36.2 ± 4.5	37.5 ± 5.8	0.429
SCP-N VD (%)	44.3 ± 2.7	44.4 ± 2.8	44.1 ± 2.4	0.720
SCP-FAZ VD (%)	11.7 ± 2.0	11.6 ± 2.0	12.2 ± 1.7	0.113
SCP-C VD^b^ (%)	56.3 ± 7.6	55.9 ± 8.1	57.1 ± 6.4	0.802
**Mean vascular density of DCP**				
Total DCP VD (%)	42.8 ± 1.7	42.7 ± 1.8	42.8 ± 1.7	0.903
DCP-A VD (%)	40.3 ± 2.8	40.1 ± 2.9	40.9 ± 2.5	0.172
DCP-N VD (%)	44.9 ± 1.9	44.9 ± 1.8	44.9 ± 2.1	0.807
DCP-FAZ VD (%)	15.7 ± 5.7	15.2 ± 5.5	17.1 ± 6.1	0.276
DCP-C VD (%)	52.2 ± 4.3	51.8 ± 4.4	53.2 ± 4.0	0.183
**Mean retinal thickness**				
Total MRT (μm)	255.8 ± 25.6	253.2 ± 25.3	262.3 ± 25.7	0.195
SCP-A MRT (μm)	233.0 ± 39.1	**226.7 ± 38.2**	**248.4 ± 37.9**	**0.022**
SCP-N MRT (μm)	265.4 ± 23.7	264.2 ± 23.2	268.5 ± 25.2	0.670
SCP-C MRT^b^ (μm)	263.0 ± 40.5	**254.3 ± 34.1**	**283.7 ± 47.4**	**0.011**
DCP-A MRT (μm)	241.1 ± 38.2	**234.6 ± 36.7**	**257.1 ± 37.9**	**0.015**
DCP-N MRT (μm)	265.4 ± 22.8	265.1 ± 22.9	266.2 ± 23.0	0.995
DCP-C MRT (μm)	264.8 ± 30.2	**259.5 ± 28.0**	**277.7 ± 32.1**	**0.006**

*SCP* superficial capillary plexus, *VD* vascular density, *SCP-A* abnormal region of superficial capillary plexus, *SCP-N* normal region of superficial capillary plexus, *FAZ* fovea avascular zone, *SCP-FAZ* foveal avascular zone of superficial capillary plexus, *SCP-C* capillary congestion of superficial capillary plexus, *DCP* deep capillary plexus, *DCP-A* abnormal region of deep capillary plexus, *DCP-N* normal region of deep capillary plexus, *DCP-FAZ* foveal avascular zone of deep capillary plexus, *DCP-C* capillary congestion of deep capillary plexus, *MRT* mean retinal thickness.

Bold font indicates a statistically significant difference (a p value < 0.05) between the non-recurrence and recurrence groups.

^a^All values are the results of the Mann–Whitney *U* test.

^b^Statistical values for 67 eyes (47 eyes in the recurrence group and 20 eyes in the non-recurrence group) in which SCP-C was observed.

The MRT of SCP-A was lower than that of SCP-N (233.0 vs. 265.4 μm, p < 0.001) in all eyes. The MRT of DCP-A was also lower than that of DCP-N (241.1 vs. 265.4 μm, p < 0.001). The MRTs of SCP-A and DCP-A were greater in the recurrence group (p = 0.022 and p = 0.015, respectively). The MRT of DCP-C did not differ from the MRT of DCP-N but was greater than that of DCP-A (p < 0.001) in all eyes. The MRTs of SCP-C and DCP-C in the recurrence group were greater than those in the non-recurrence group (p = 0.011 and p = 0.006, respectively).

### Risk factors for the recurrence of macular edema in chronic stable BRVO

The recurrence group relapsed after a mean of 7.2 ± 2.6 months from baseline, and CSMT increased to 362.2 μm from baseline (p < 0.001). Among baseline characteristics and OCTA parameters, univariate logistic regression analysis revealed old age, a long period from initial visit to baseline, a higher number of injections, and a higher MRT of SCP-A, DCP-A, and DCP-C as risk factors for the relapse of ME (Table 4). In multivariate logistic regression analysis, only the number of intravitreal injections (odds ratio [OR]: 1.803 [95% confidence interval {CI} 1.329–2.446]; p < 0.001) and the MRT of DCP-C (OR: 1.044 [95% CI 1.017–1.073]; p = 0.002) remained significant predictors of recurrence of ME.

**Table 4:** Logistic regression analysis of the recurrence of macular edema.

	Univariate logistic regression analysis		Multivariate logistic regression analysis	
	Odds ratio (95% CI)	p value^a^	Odds ratio (95% CI)	p value^a^
Age (years)	1.056 (1.005–1.110)	0.031	–	–
Period from initial visit to baseline (months)	1.173 (1.065–1.292)	0.001	–	–
Total number of injections (N)	1.479 (1.193–1.834)	< 0.001	1.803 (1.329–2.446)	< 0.001
Number of anti-VEGF injections (N)	1.435 (1.116–1.845)	0.005	–	–
Number of steroid injections (N)	1.592 (1.075–2.357)	0.020	–	–
SCP-A MRT (μm)	1.015 (1.001–1.029)	0.032	–	–
DCP-A MRT (μm)	1.016 (1.002–1.031)	0.023	–	–
DCP-C MRT (μm)	1.022 (1.003–1.040)	0.021	1.044 (1.017–1.073)	0.002

*CI* confidence interval, *VEGF* vascular endothelial growth factor, *SCP-A* superficial capillary plexus-abnormal vessel area, *MRT* mean retinal thickness, *DCP-A* deep capillary plexus-abnormal vessel area, *DCP-C* deep capillary plexus-congestion area.

^a^All values are the results of logistic regression analysis.

### Correlation between mean retinal thickness of the capillary congestion and vascular densities of the segmented regions

The MRT of SCP-C was significantly positively correlated with the mean VDs of SCP-A (Spearman's correlation coefficient r_s_ = 0.318; p = 0.009) and DCP-A (r_s_ = 0.305; p = 0.012). The MRT of DCP-C was positively correlated with the mean VDs of SCP-N (r_s_ = 0.355; p = 0.002), SCP-A (r_s_ = 0.427; p < 0.001), DCP-N (r_s_ = 0.365; p = 0.001), DCP-A (r_s_ = 0.470; p < 0.001), and DCP-C (r_s_ = 0.321; p = 0.005).

### Spatial distribution between deep capillary plexus congestion and macular edema

**Figure 4:**
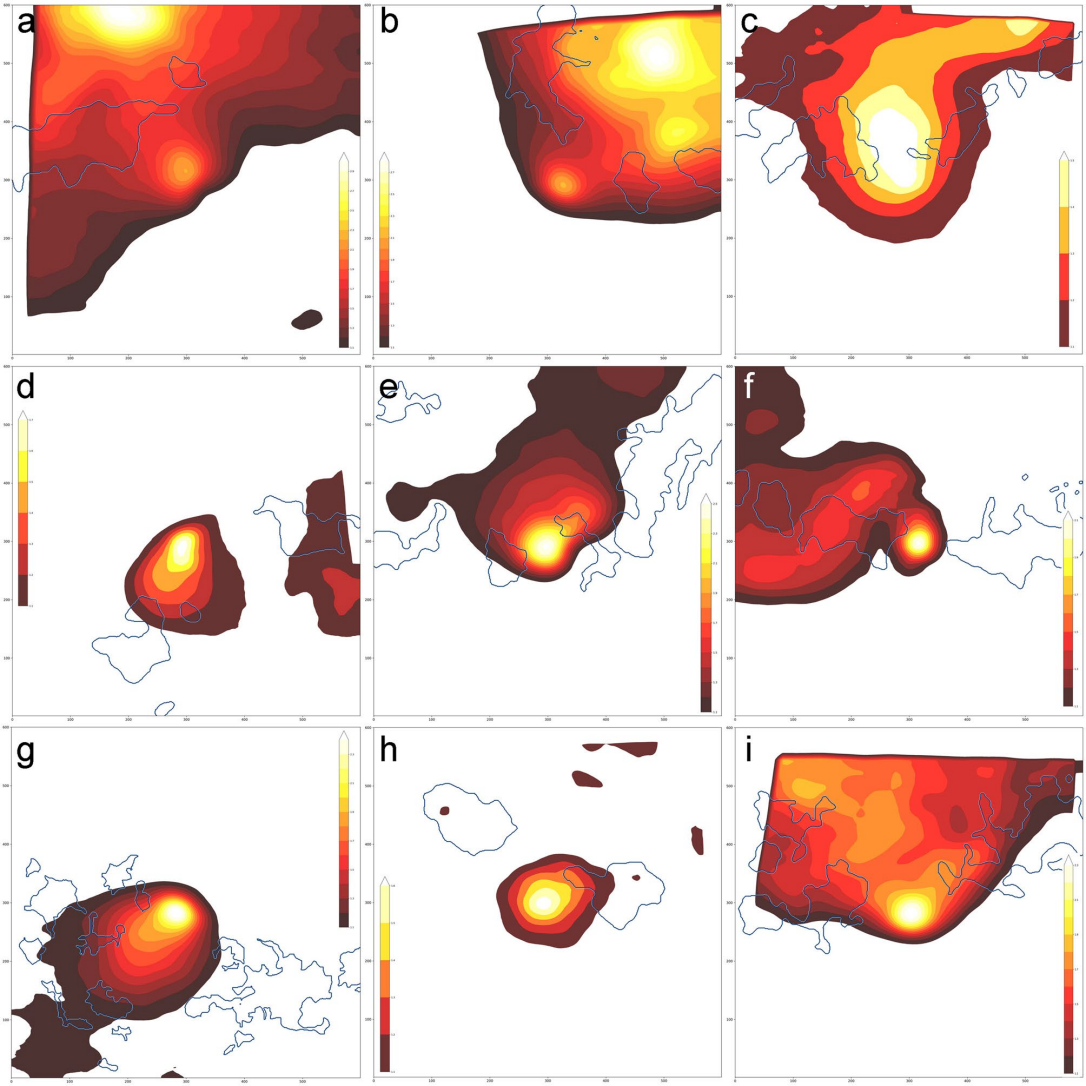
Contour plots of the territory of macular edema with boundaries of deep capillary congestion at recurrence. Patients with chronic stable branch retinal vein occlusion experienced recurrence of macular edema (ME) from baseline. (**a**–**c**) Apexes of the ratio of an increased retinal thickness (RIRT) were in the capillary nonperfusion area (NPA) of the affected side. The other local maximums were in the foveal avascular zone (FAZ). Capillary congestion of the deep capillary plexus (DCP-C) surrounded the local apexes of RIRT. (**d**–**f**) Unlike the cases depicted in (**a**–**c**), the apexes of RIRT existed in the FAZ. Some DCP-C segments were not involved in ME. (**g**–**i**) Similar to the cases depicted in (**d**–**f**), the apexes of RIRT were in the FAZ. Most DCP-Cs existed on the border of ME territories, and in other DCP-Cs (**h**), ME has just emerged.

The mean RIRT of the ME territories between recurrence and baseline was 1.326 ± 0.174 and was higher than that of DCP-C (1.084 ± 0.097, p < 0.001). In the recurrence group, the apexes of the ME territories were located at the FAZ (16 eyes, 72.7%) or the NPA (6 eyes, 27.3%) and were juxtaposed with or surrounded by DCP-C (Fig. 4). The maximum RIRT of the ME territory was always higher than that of DCP-C (1.984 ± 0.566 vs. 1.568 ± 0.429, p < 0.001).

## Discussion

Macular cyst formation, absent collateral vessels, microaneurysms, disorganization of the inner retinal layer, and increased gap vessel area are known as microvascular abnormalities involved in the ME in BRVO^11,19,24,25^. Our study revealed increased thickness of DCP-C as the only risk factor for the recurrence of ME among various OCTA parameters in eyes with chronic BRVO. The risk increased by 4.4% with increasing MRT of DCP-C by 1.0 μm; briefly, a 17 μm increase in MRT of DCP-C doubled the risk. A subtle change in thickness of DCP-C may be associated with a relapse, implying that hidden mechanisms for aggravating or suppressing ME preexist in the DCP-C region.

About three-fifths of DCP-C occupied the TZ in chronic stable BRVO. DCP-C had more abundant flow (higher VD) than normal capillary plexuses; the congestion could arise when stagnant flows of affected venules bypass the DCP in the TZ and then drain to the unaffected side. Campbell et al. proposed the hammock structure of the retinal capillary plexus^26^. Unlike the upper capillary networks, the DCP is known to form a collateral circulation with spider-like vortex capillaries that traverse the horizontal raphe^27^. In the normal macula, the arteriolar flow into the DCP is drained to the adjacent venule or the distant venules through vortex capillaries; these capillaries of the DCP seem to be physiological pathways to remove deoxygenated blood cells passing through the outer plexiform layer demanding high oxygen^27^. In acute BRVO, the capillary flow can hardly drain into the affected venule with high intraluminal pressure, crosses the transverse vortex capillaries, and finally drains into the distant DCP and venules of the unaffected sides. At the DCP near the TZ, combined venous flows from the unaffected and affected sides can lead to high VD and dilation of the capillary walls, similar to how we defined DCP-C. After the acute phase of BRVO, OCTA can depict capillary congestion as tortuous and dilated angioflow signals predominantly in the DCP^17^. DCP-C is considered a physiological adaptation to local flow regulation against increased hemodynamic stress and pressure gradient^28^. In addition to all patients in our study who had chronic stable BRVO, this phenomenon has been reported not only in other BRVO patients but also in animal models of BRVO^11,13,29,30^.

Normal DCP, with its lower hydrostatic pressure and slower flow speed than those in normal SCP, is thought to be a primary pathway for absorption of interstitial fluids^31^. However, DCP-C in BRVO exhibits faster and greater flows with higher hydrostatic pressure, so its absorptive function may be degraded compared with that of normal DCP. At baseline, the MRT of DCP-C is positively correlated with not only the normal regions of VDs but also the abnormal regions of VDs. Like a sponge holding water, the DCP-C region can become thicker as the flow increases before anatomical abnormalities present as intraretinal or subretinal fluids on the structural OCT.

Several studies reported atypical leakage in the capillary congestion zone (i.e., collateral vessels) of BRVO^14,16^. Unlike retinal neovascularization, capillary congestion can leak in the late phase of FA^14^. The leakage may or may not be accompanied by aneurysmal dilatation^16^. Maturation of capillary congestion occurs 6–24 months after the onset of BRVO, and transient retinal edema has also been found during this period^14^. Capillary congestion being continuously remodeled and weakened by exceeding the hydrodynamic threshold may leak through the damaged inner blood retinal barrier (BRB) of the congested capillary wall. Capillary loss localized to the DCP was more frequently detected after anti-VEGF treatment in persistent ME with BRVO, suggesting that damage to the inner BRB of the DCP is involved in ME recurrence^19^. Leakage from the capillary wall of DCP-C spreads multidirectionally to the interstitial space, leading to accumulation of fluid in the interstitial space as reflected in various anatomic changes, such as increased retinal thickness, small cystic changes, and cystoid ME or subretinal fluids^24,32,33^. Moreover, fluids entering the interstitial space to the FAZ or NPA with absent capillary plexus of the superficial and deep layers and decreased absorptive function of Müller cells would not be easily absorbed, which would lead to continuous accumulation of fluid, resulting in ME. Therefore, thicker DCP-C is highly suspected of leaking fluid into the adjacent interstitial spaces. As illustrated in Fig. 4, DCP-C was juxtaposed with the local maxima of ME or surrounded the territories of ME. Its spatial distribution reinforces the view that DCP-C may be the source of fluid leakage.

DCP-C was observed in all eyes in this study, but ME recurred in about a third. Suzuki et al. observed leakage from capillary congestion in one-third of BRVO patients^16^. Moreover, in 70% of eyes with capillary congestion, ME resolved; however, in the remaining 30%, it did not^16^, a result that resembles our ME recurrence rate. At baseline, SCP-C was not found in all of the eyes and its area was smaller than that of DCP-C. SCP-C formation seems to be slower than the development of DCP-C due to lack of collaterals and higher net pressure caused by the shorter capillary distance in SCP^31^. Early formation of DCP-C can reduce the overall intraluminal pressure to the occluded area, and the possibilities of the growth of SCP-C may lessen consequently.

The total number of injections from the initial visit to baseline was also a risk factor for ME recurrence in chronic BRVO. According to real-world data that do not strictly control the treatment with injections, the number of injections tends to be determined by the extent of ischemia of BRVO^34,35^. In that context, in patients who required many injections, leakage from inner BRB damaged by the VEGF or inflammatory cytokines secreted from the wider ischemic lesion resulted in frequent recurrence of ME.

Except for the area and VDs of SCP-N and DCP-N, the average values were larger and higher in the recurrence group, although no statistically significant differences were noted. We only investigated a 6 × 6 mm^2^ area of the macula. Further investigation by wide-field OCTA will be required to cover broader boundaries between perfusion and nonperfusion. Highly saturated VDs on DCP-C with fast and abundant flow might result in no significant differences between the two groups, considering a sigmoid relationship between VD and flow speed^36^.

The current study had several limitations, including the small sample size and its single-center retrospective design. Selection bias might arise from the disproportion due to a smaller number of cases in the recurrence group. Various intravitreal injection regimens have been employed. Further investigation by taking FA on a chronic stable phase will be required to unravel whether leakage or aneurysmal dilatations are associated with thicker DCP-C. It is not easy to expand our results to nonischemic BRVO, because this study included only ischemic BRVO. However, considering that nonischemic BRVO has a better clinical course than ischemic BRVO and that the degree of ischemia affects various OCTA parameters^37–39^, we can reduce the confounding factors on baseline characteristics and OCTA parameters by excluding nonischemic BRVO. Prospective studies are needed to determine whether early treatment or shortening the follow-up period according to the thickness of DCP-C reduces the ME recurrence rate. Nonetheless, noninvasive assessment of DCP-C using en-face OCTA revealed the decisive clinical findings for the recurrence of ME from chronic stable BRVO. With en-face OCTA and segmented data from this study, the basis for automatic segmentation for capillary congestion or abnormal vascular lesions was prepared for the future application of artificial intelligence. This future work will provide the MRT of DCP-C easier and faster to the ophthalmologist.

In conclusion, DCP-C existed in all eyes with chronic stable BRVO, and increased retinal thickness of DCP-C was an important OCTA finding to predict the recurrence of ME from chronic stable BRVO. DCP-C seems to provide a drainage pathway against elevated intravenous pressure, which may sustain the stability of chronic BRVO, but it may simultaneously be the source of ME.

## Supplementary Information

Supplementary Figure S1.

Supplementary Figure S2.

Supplementary Figure S3.

Supplementary Table S1.

## Data Availability

Data are available upon reasonable request.
