# Supplementary material for: Association between capillary congestion and macular edema recurrence in chronic branch retinal vein occlusion through quantitative analysis of OCT angiography
Source: Sci Rep. 2021 Oct 6;11:19886. doi: 10.1038/s41598-021-99429-z (PMC8494742; doi:10.1038/s41598-021-99429-z)
Supplement: Supplementary file 1 — Supplementary Figure S1. [file 41598_2021_99429_MOESM1_ESM.docx]

**Supplementary Figure S1. Schematic flow chart of the study design.**

**
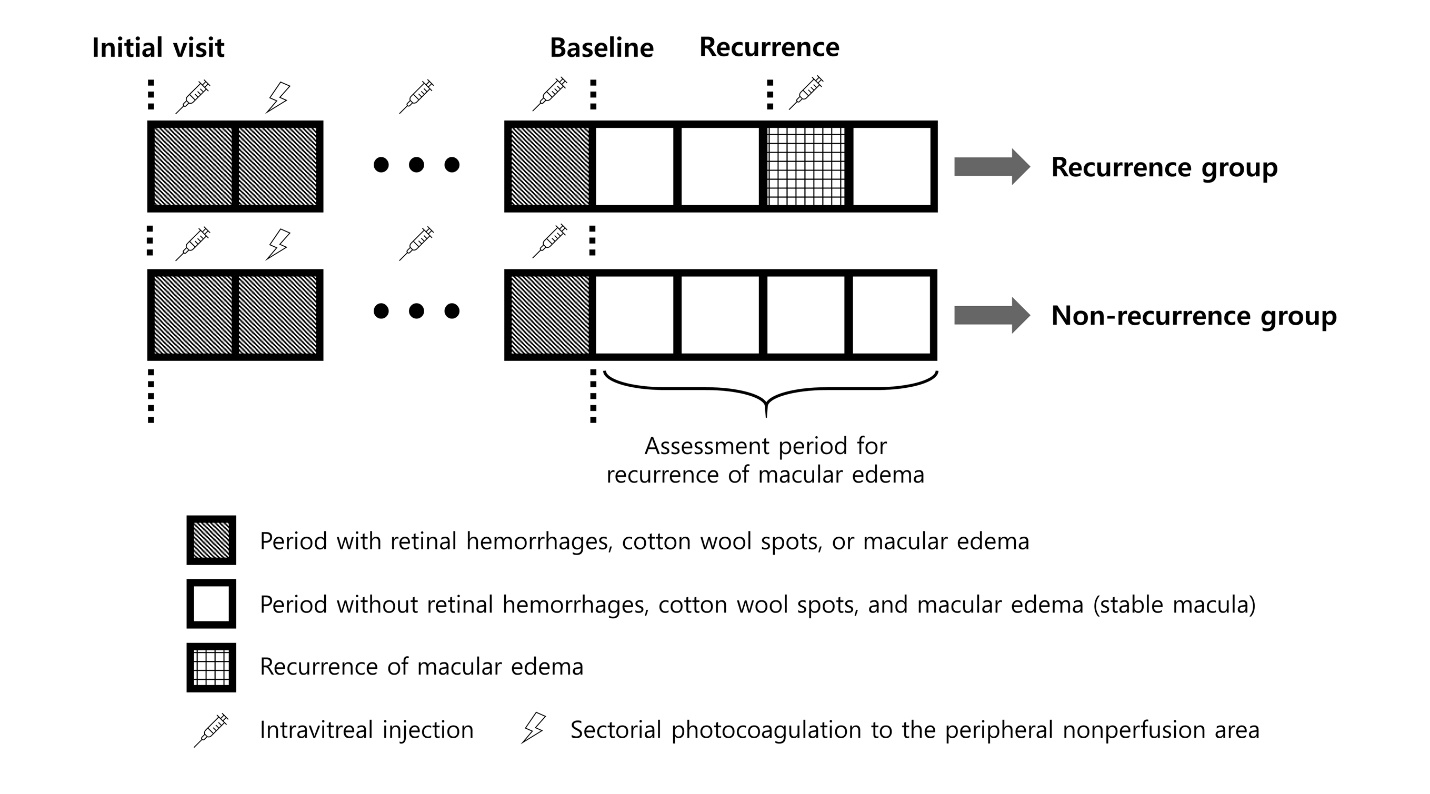
**

This study included patients with major and ischemic branch retinal vein occlusion who received acute phase treatments (left diagonal patterned box) and exhibited stable macula (white box) for at least two consecutive visits with a 3-month interval from 6 months after the initial visit. The first visit in the period of the chronic stable macula was set as the baseline. From the baseline to 12 months, recurrence of macular edema (checkered box) was assessed with fovea-centered 6 x 6 mm^2^ macular volume scans and defined as an increase in central subfield macular thickness by 10% or more due to fluids.
