# Supplementary material for: Association between capillary congestion and macular edema recurrence in chronic branch retinal vein occlusion through quantitative analysis of OCT angiography
Source: Sci Rep. 2021 Oct 6;11:19886. doi: 10.1038/s41598-021-99429-z (PMC8494742; doi:10.1038/s41598-021-99429-z)
Supplement: Supplementary file 2 — Supplementary Figure S2. [file 41598_2021_99429_MOESM2_ESM.docx]

**Supplementary Figure S2. Conversion of color-coded vessel density and retinal thickness map.**

**
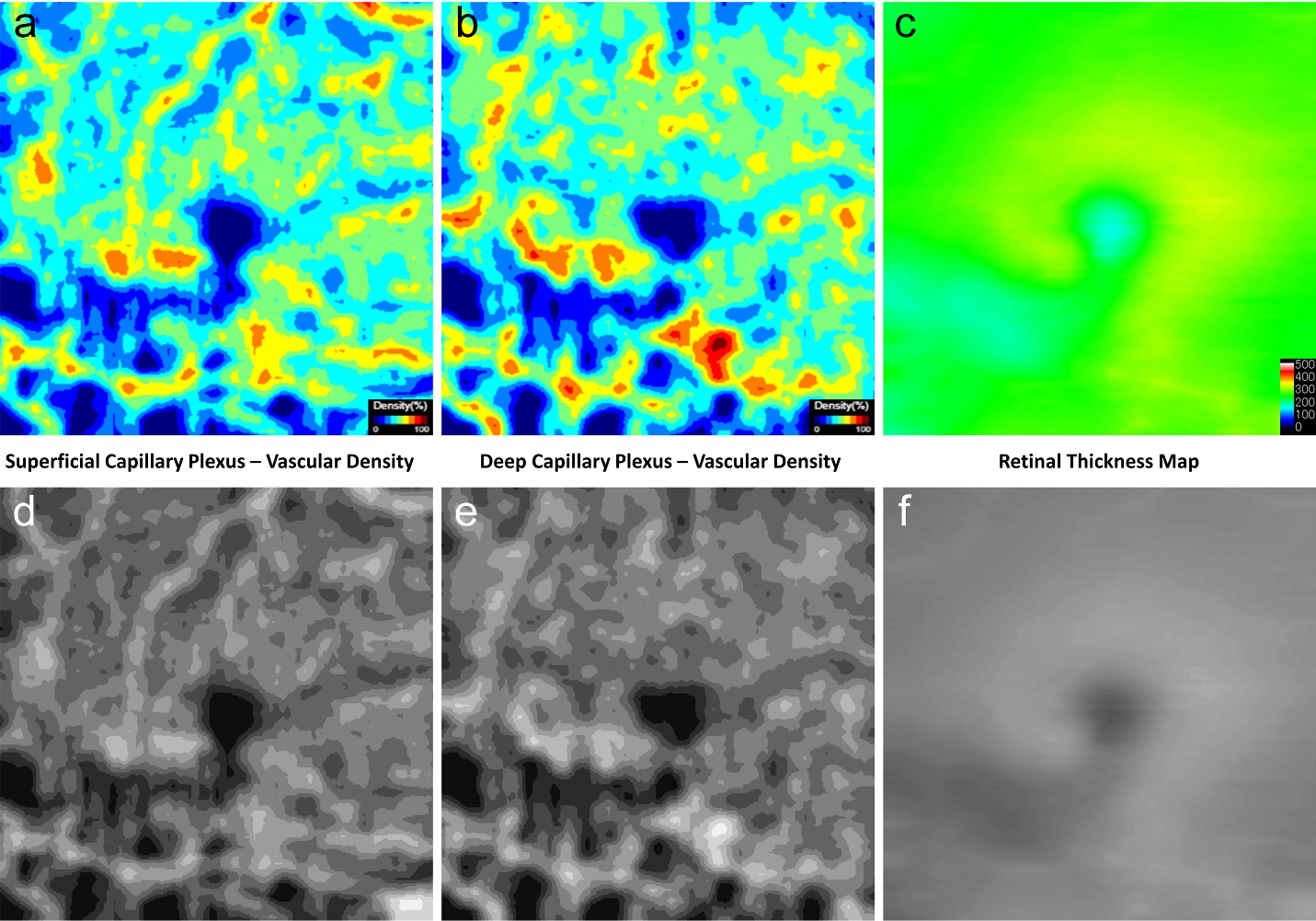
**

(**a, b**) Color-coded vascular density (VD) maps of superficial (**a**) and deep (**b**) capillary plexuses of the same scan area shown in **Fig. 1**. The color-code bar is exposed at the bottom right of each image. (**c**) The color-coded retinal thickness map of the same region. The VD map's color code varies from 0 to 100%, and the color-code of the retinal thickness map varies in the range of 0 to 500 μm. (**d ~ f**) Each pixel's color information of the upper row image was converted into a 16-bit gray-scale image for calculating the average value of the segmentation region. The pixel data of the gray-scale image itself indicate the VD or retinal thickness values.
