# Supplementary material for: Association between capillary congestion and macular edema recurrence in chronic branch retinal vein occlusion through quantitative analysis of OCT angiography
Source: Sci Rep. 2021 Oct 6;11:19886. doi: 10.1038/s41598-021-99429-z (PMC8494742; doi:10.1038/s41598-021-99429-z)
Supplement: Supplementary file 3 — Supplementary Figure S3. [file 41598_2021_99429_MOESM3_ESM.docx]

**Supplementary Figure S3. Representative cases of the non-recurrence and recurrence group.**

**
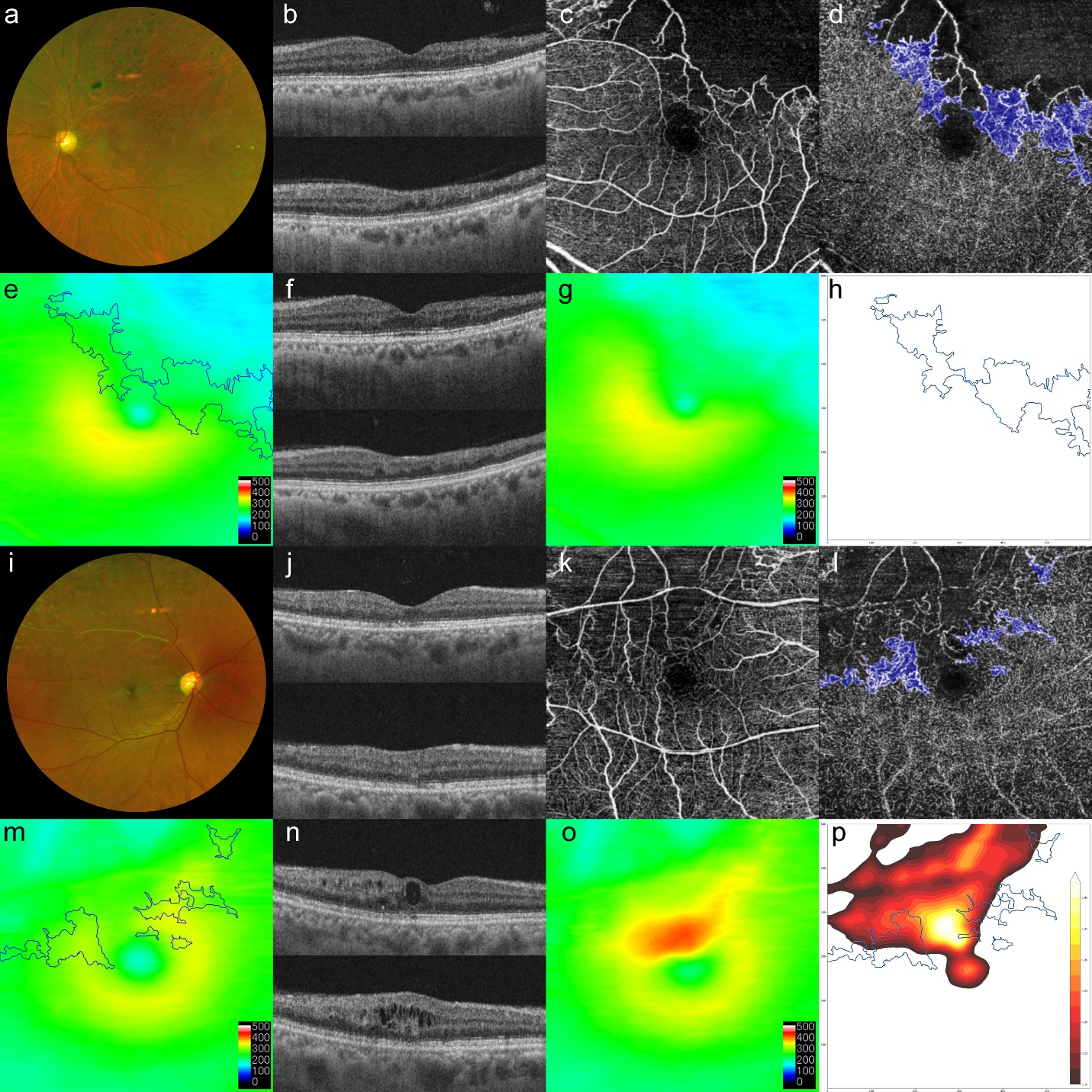
**

(**a~h**) A representative case of the non-recurrence group. A 56-year-old male with left major ischemic branch retinal vein occlusion (BRVO) reached (**a**) stable macula at 10.8 months from the initial visit after two anti-vascular endothelial growth factor (VEGF) injections. (**b**) In horizontal B-scan optical coherence tomography (OCT) passing through the fovea (upper) and 1 mm above the fovea (lower), macular edema (ME) was absent. The central subfield macular thickness (CSMT) was measured to be 232 μm. (**c~d**) Superficial and deep capillary plexus (SCP and DCP) images of en-face OCT angiography (OCTA) showed abnormal vascular and capillary congestive regions of DCP (DCP-C, blue region). (**e**) The color-coded retinal thickness map with the color code (bottom left) provided by the manufacturer presents absent ME. Mean retinal thickness (MRT) of the DCP-C (area surrounded by blue lines) was calculated to be 216.2 μm. (**f~g**) No recurrence was noted on the same regions of **b** and **e** at 1 year. (**h**) After analysis of the ratio of an increased retinal thickness (RIRT) from the baseline to 1-year, retinal thickness did not increase more than 10% at any point. (**i~p**) A representative case of the recurrence group. A 49-year-old male with right major ischemic BRVO reached (**i**) stable macula at 16.3 months from the initial visit after four anti-VEGF injections. (**j**) CSMT was 235 μm without ME at the baseline. (**k~l**) Abnormal lesions and DCP-C (blue in **l**) were detected. (**m**) MRT of the DCP-C was measured to be 306.6 μm. (**n**) Six months later, CSMT increased to 310 μm due to ME recurrence. (**o**) Detailed ME territory was revealed at recurrence. (**p**) The apex of RIRT was located above the fovea and surrounded by DCP-C. Another local maximum of RIRT was positioned at the foveal avascular zone and connected with the apex of RIRT.
