# Supplementary material for: Association between capillary congestion and macular edema recurrence in chronic branch retinal vein occlusion through quantitative analysis of OCT angiography
Source: Sci Rep. 2021 Oct 6;11:19886. doi: 10.1038/s41598-021-99429-z (PMC8494742; doi:10.1038/s41598-021-99429-z)
Supplement: Supplementary file 4 — Supplementary Table S1. [file 41598_2021_99429_MOESM4_ESM.docx]

**Supplementary Table S1. Exclusion criteria of the patients with chronic branch retinal vein occlusion**

| Exclusion Criteria | Number of Patients (N) |
| --- | --- |
| Major BRVO without invasion of the macular region | 4 |
| Attenuation of the ellipsoid zone after acute-phase treatments | 3 |
| New occlusion of other retinal veins after the initial visit | 3 |
| BRVO caused by retinal vasculitis | 2 |
| BRVO co-occurring with branch retinal artery obstruction | 3 |
| BRVO co-occurring with ocular ischemic syndrome | 1 |
| Diabetic retinopathy | 5 |
| Presence of epiretinal membrane presenting loss of the foveal depression | 2 |
| High myopia (an axial length ≥ 26.5 mm or a spherical equivalent ≥ − 6.0 diopters) | 3 |
| Previous vitrectomy | 4 |
| Previous filtering surgery | 2 |
| Overlapping retinal vessels provoked by eye movement or tracking error | 5 |
| Poor OCTA image quality (less than 60 points) | 2 |
| **Total** | 39 |

BRVO = Branch retinal vein occlusion; OCTA = Optical coherence tomographic angiography.
